# Vitamin D in Neurological Diseases: A Rationale for a Pathogenic Impact

**DOI:** 10.3390/ijms19082245

**Published:** 2018-07-31

**Authors:** Rita Moretti, Maria Elisa Morelli, Paola Caruso

**Affiliations:** Neurology Clinic, Department of Medical, Surgical and Health Sciences, University of Trieste, Strada di Fiume, 447, 34149 Trieste, Italy; mariaelisa.morelli@gmail.com (M.E.M.); caruso.paola1983@libero.it (P.C.)

**Keywords:** neuro-degeneration, MS, demyelination, vascular disease, stroke, AD, vitamin D-OH 25, VDR, VDH, calcium

## Abstract

It is widely known that vitamin D receptors have been found in neurons and glial cells, and their highest expression is in the hippocampus, hypothalamus, thalamus and subcortical grey nuclei, and substantia nigra. Vitamin D helps the regulation of neurotrophin, neural differentiation, and maturation, through the control operation of growing factors synthesis (i.e., neural growth factor [NGF] and glial cell line-derived growth factor (GDNF), the trafficking of the septohippocampal pathway, and the control of the synthesis process of different neuromodulators (such as acetylcholine [Ach], dopamine [DA], and gamma-aminobutyric [GABA]). Based on these assumptions, we have written this review to summarize the potential role of vitamin D in neurological pathologies. This work could be titanic and the results might have been very fuzzy and even incoherent had we not conjectured to taper our first intentions and devoted our interests towards three mainstreams, demyelinating pathologies, vascular syndromes, and neurodegeneration. As a result of the lack of useful therapeutic options, apart from the disease-modifying strategies, the role of different risk factors should be investigated in neurology, as their correction may lead to the improvement of the cerebral conditions. We have explored the relationships between the gene-environmental influence and long-term vitamin D deficiency, as a risk factor for the development of different types of neurological disorders, along with the role and the rationale of therapeutic trials with vitamin D implementation.

## 1. Introduction

Low levels of vitamin D, considering serum 25-hydroxyvitamin D (25(OH)D), have been recognized as a widespread health problem, affecting approximately one billion people worldwide [1]. Latitude, season, cultural norms, religious practices, limited awareness, lack of knowledge, indoor lifestyles, urban living, skin pigmentation, malnutrition, diet, co-morbidities (like tuberculosis, malnutrition, chronic inflammatory conditions, etc.), and drugs, may contribute to vitamin D deficiency, especially in the developing world, even if some genetic predispositions can interfere with it (i.e., blacks tend to have lower levels of 25(OH)D compared to whites) [2,3].

Vitamin D is a lipid-soluble vitamin, which can be synthesized and act as a hormone [4]. The active form of vitamin D, 1,25(OH)_2_D, known as calcitriol, has chemical similarities to typical hormones, such as testosterone, estrogen, and cortisol.

Vitamin D yields both genomic and non-genomic actions; the Vitamin D Receptors (VDR) mediates the former, one of the representative of the steroid hormone superfamily, which are evident in more than 30 human tissues, therefore regulating 3% of the human genome (approximately 700 genes) [5]. Nuclear VDRs are found in most cells, and support the role for the extra-skeletal benefits of vitamin D. The VDR are found in almost all human tissues, participating in the classic actions of vitamin D in the bone, gut, and kidney, but are also involved in immune functions, hormone secretion, and cellular proliferation and differentiation [6]. To be biologically active, vitamin D undergoes hydroxylations in the liver, mediated by the 25-hydroxylase, and in the kidney, mediated by 1α-hydroxylase. The 1,25(OH)_2_D is recognized by VDR, in various cells, mainly in the intestine. As vitamin D plays a significant role in modulating the immune system in the intestine, it is possible that its deficiency could deteriorate the gut barrier function, favoring the translocation of endotoxins. Vitamin D deficiency has been associated with intestinal dysbiosis and increased susceptibility to intestinal diseases; moreover, vitamin D could be contributing to disturbances of the glucose metabolism by modulating the composition of the gut microbiota. The changed intestinal microbiome has become epidemic, in parallel with the epidemic of vitamin D deficiency, suggesting that they might be linked. Proper supplemental doses of vitamin D plus all of the B vitamins appears to return the intestinal microbiome back to normal after few months [7,8]. Moreover, there are more and more evidence that links the microbiome to neurologic disorders [9], which can share a common auto-immune involvement; the potential link between the three actors (VDR, neurological disorders, and vitamin D) is quite fascinating and many studies should be done to gain knowledge on them.

The non-genomic actions of vitamin D help to cooperate with the classical genomic pathway, to transactivate VDR, and exert the effects of calcitriol; the signal does not depend on the transcription phases, but it can operate via cross-talk with different signaling pathways, such as rapid membrane response binding proteins (see later). The genomic and the non-genomic pathways support the emerging not calcemic effects of the vitamin D metabolites, trying to define a role in the autoimmune pathologies, infectious diseases, diabetes mellitus, obesity, and cardio-metabolic disorders [10].

It has also been demonstrated that Vitamin D is involved in different neurological disorders, such as multiple sclerosis, Alzheimer’s disease, Parkinson’s disease, and stroke [11,12,13,14].

The presence of VDR in the hippocampus, hypothalamus, thalamus, cortex, and substantia nigra [15] prompted many studies on the possible determinant role of vitamin D in different neurological conditions [16,17]. It has been evidenced that calcitriol is a fundamental actor in the neuronal differentiation and the neural maturation [18]. Vitamin D normalizes the trafficking of the septo-hippocampal pathways, mainly via the neural growth factor (NGF); moreover, it is an active controller of the genetic regulation of the synthesis of acetylcholine (Ach), dopamine (DA), serotonin (5HT3), and gamma-aminobutyric (GABA) [19,20,21].

The knock-out model of VDR^−/−^ has an accelerated aging process in all of the organs, as well as in the brain [22,23], with a significant in-brain decrease of NGF [24] and other neurotransmitters, such as Ach [25,26]. The congenital deficiency of vitamin D significantly reduces the activity of glutamic acid decarboxylase (GAD) 65/67 (critical enzymes in GABAergic inter-neurons) and the levels of glutamate and glutamine in the brain tissue [27]. 

For these reasons, we reckon that a vitamin D deficiency could contribute to the complex relationship between genetics and the environment, the two common poles that many neurological pathologies debate between. It could intervene in the exacerbation of precipitating factors or in demodulating the repair process; therefore, we consider that this topic should be approached from a neurological perspective. Therefore, we have started this review. We searched MEDLINE using the following search terms: “vitamin D central nervous system”, both “vitamin D” and “central nervous system”; “vitamin D immune system/response”, both “vitamin D” and “immune system” or “immune response”; “vitamin D multiple sclerosis risk”, both “vitamin D” and “multiple sclerosis risk”; “vitamin D multiple sclerosis relapse”, both “vitamin D” and “multiple sclerosis relapse”; “vitamin D multiple sclerosis magnetic resonance imaging”, both “vitamin D” and “multiple sclerosis magnetic resonance imaging”; “vitamin D multiple sclerosis disability”, both “vitamin D” and “multiple sclerosis disability”; “vitamin D supplementation/therapy/treatment multiple sclerosis”, both “vitamin D supplementation” or “vitamin D therapy” or “vitamin D treatment” and “multiple sclerosis”; “vascular dementia”, both “vascular” and “dementia”; “subcortical vascular dementia”, both “subcortical” and “dementia”; “Alzheimer’s disease”; “pathogenesis neural-degeneration”; “amyloid”; “cholinergic afferents”; “arteriolosclerosis”; “cerebral flow regulation”; and “stroke”. The publications that were selected were mostly from the past 20 years, but they did not exclude the frequently referenced and highly regarded older publications. The research has been extended, with the same strings, EMBASE, COCHRANE LIBRARY, and LILACS. All of the searches were done from 1 January 1993 up to 31 May 2018. We considered papers published in English, French, German, and Italian. Congress abstracts and isolated case reports (even if the total cases were under 10) were not considered. Review articles and book chapters are cited to provide additional details. The authors carefully read all of the selected articles.

## 2. Vitamin D Deficiency and Multiple Sclerosis: Role in the Susceptibility, Activity, and Treatment of the Disease

Multiple Sclerosis (MS) is a multifactorial demyelinating pathology affecting the brain and the spinal cord, firmly relying on an altered immune response. Activated autoreactive T cells invade the blood–brain barrier and initiate an inflammatory response that leads to myelin destruction and axonal loss. The etiology of MS, the mechanisms associated with its onset, the unpredictable clinical course, and the different rates of progression leading to disability over time, remain unresolved questions [28].

Immunological research makes two significant observations to explain the link between vitamin D and the immune system.

Firstly, most immune cells, of both the innate and the adaptive immune system, express the VDR [29,30]. Secondly, the immune cells exhibit an active vitamin D metabolism, with the expression of the rate-limiting enzyme for vitamin D synthesis, 1α-hydroxylase (CYP27B1) [31]. Immune cells are, therefore, able to synthesize and secrete vitamin D in an autocrine and paracrine condition [32]. The immune cell types, targeted by vitamin D, include monocytes and macrophages, dendritic cells (DCs), and T and B cells [33]. 1,25(OH)_2_ vitamin D induces monocytes proliferation and differentiation into macrophages [34], the potentiate the expression of interleukin-1 (IL-1), and the antimicrobial peptides (cathelicidin, β-defensin-2, and hepcidin) [35]. Vitamin D inhibits DC differentiation and maturation, their major histocompatibility complex (class II) expression (MHC), CD40, CD80, CD86, and IL-12 (while inducing the production of IL-10), leading to reduced T cell stimulatory capacity [36,37]. Vitamin D decreases the production of nitric oxide (NO), mediating the downregulation of the inducible nitric oxide synthase (iNOS) expression [38]. Moreover, it stimulates the development of natural killer T (NKT) cells, and it increases IL-4 and interferon (IFN)-γ production [39]; vitamin D attenuates the proliferation of CD8+ T cells and reduces their cytotoxic activity by decreasing the production of IL-2, IL-17, and IFN-γ.

Vitamin D exerts its immunomodulatory effects on T lymphocytes, by inhibiting the production of pro-inflammatory Th1 cytokines (IL-1, IL-2, IL-6, IL-12, IFN-γ, TNF-α, and TNF-β), and stimulating the production of anti-inflammatory regulatory Th2 cytokines (IL-4, IL-5, and IL-10) [40,41].

Thus, vitamin D potentiates the innate immune system and regulates the adaptive immune system, mainly by inducing the split to Th2 and regulatory T cells (Tregs), over the Th1 and Th17 lymphocytes differentiation [42], via direct and indirect actions on naive CD4+ cells. The general result is an evident effect of switching capacity, from a pro-inflammatory autoimmune to an anti-inflammatory tolerogenic immunological response.

In multiple sclerosis (MS) patients, the blood level of 25(OH)D or 1,25(OH)_2_D has been linked with the suppressive activity of Tregs [43], and the number of Tregs has been correlated with the serum levels of 25(OH)D or 1,25(OH)_2_D. The Tregs are increased in the MS patients that have supplemented with vitamin D [44,45].

According to many studies, vitamin D may have an impact on the balance between the inflammatory and anti-inflammatory mechanisms, which might help/regulate the remyelination process. Vitamin D increases the microglial activation, promoting the clearance of myelin debris, and consequently activating the remyelination process [46]. In the oligodendrocyte precursor cells (OPC) cultures, vitamin D up-regulates the transcription of VDR and NGF mRNA, but not of myelin basic protein (MBP) [47,48,49]. The remyelination of demyelinated lesions has been observed in the early stages of MS [50,51], and it has been supported by neuroimaging findings [52,53]. However, remyelination might be incomplete [54], and eventually, it might cease [55], because of the OPC inability to migrate and reach the site of demyelination [56], or for the OPC differentiation inability [57]. In fact, an inflammatory microenvironment prevents OPC maturation and the differentiation into oligodendrocytes, and subsequently prevents axon remyelination. This is relevant for MS treatment, as current treatments are only useful for controlling immune mechanisms (i.e., in the early stages of the disease), but do not affect remyelination. In vitro studies show that when blocking VDR, there is a reduction of OPC differentiation, with a consequent blockage of myelination and remyelination; on the other hand, by activating VDR, via vitamin D, there is an increase in the differentiation of OPC and the consequent remyelination [58]. Likewise, neural stem cells (NSC) express VDR and 1,25(OH)_2_D, which seems to promote the NCS proliferation and differentiation into neurons and oligodendrocytes, and to reduce astrogliosis [59,60].

As vitamin D deficiency has been proposed as a significant risk factor in MS development, most epidemiologic observational studies have suggested that adequate vitamin D levels may reduce the risk of MS onset and modify the course of the disease. MS is a disease that is virtually unknown at the equator, and the prevalence of the disease increases in populations that live farther away from the equator [61]. The prevalence of MS is higher at higher latitudes, and tends to peak in the areas with the lowest exposure to ultraviolet (UV) light [62,63]; however, in these areas, diets rich in vitamin D-containing oily fish may offset this risk to some degree [62,63,64,65]. Moreover, the risk of MS has been found to decrease among people who migrate from higher to lower latitudes [66]. This latitudinal finding has been declining in recent decades, instead of an associated increasing trend towards avoiding sun exposure by staying indoors for more extended periods of the day, even in warmer climates [67,68]. In fact, higher levels of sun exposure (past, recent, and cumulative) were independently associated with higher levels of vitamin D and with a significantly reduced risk of developing demyelinating events [69]. Sunlight seems to have an immunosuppressive effect and, therefore, the effects of sunlight on MS risk could be related to the sunlight itself, or to an increase of vitamin D [70]. The association between the calculated vitamin D intake from diet or supplements, and the risk of developing MS has been prospectively evaluated in two large cohorts involving more than 187,000 women [71]. Women who had a higher intake of dietary vitamin D (approximately 700 IU/day) had a 33% lower incidence of MS compared with those with a lower intake. Moreover, the women who used vitamin D supplements (more than 400 UI/day) had a 41% reduced risk of developing MS compared with non-users. Higher levels of 25(OH)D (independently, from dietary vitamin D intake) also seem to predict a lower risk of MS onset. A more recent prospective study confirmed these findings and reported that the levels of vitamin D over 30 ng/mL were associated with a decreased MS risk [72]. Adiposity has been associated with lower vitamin D levels [73,74], and a higher body mass index (BMI) has been associated with higher incidence of MS in adolescent women, but not in adult women [75].

A crucial question related to a primary prevention trial or from vitamin D supplementation in MS is the relevant age of exposure, which can range from in utero to adolescence and adulthood [76]. Mirzaei et al. studied a large cohort and analyzed the association between maternal dietary vitamin D intake and the predicted maternal serum 25(OH)D during pregnancy, and their daughters’ risk of developing MS [77]. The study showed that the relative risk of MS was significantly lower in the women whose mothers had high vitamin D intake during pregnancy compared with the women born to low-intake mothers. A diminished in utero exposure to vitamin D, coupled with the solar cycle and latitudinal differences, may be an environmental risk factor for the development of MS. Similarly, albeit not statistically significant, a reduced MS risk was reported among the women reporting an increased vitamin D intake from supplements in adolescence [78]. These results suggest that MS risk is related not only to new vitamin D levels, but it might also be related to its levels during childhood or even in utero. Several studies, including a meta-analysis, demonstrated that spring-born have a significantly higher lifetime MS risk than autumn-born, which has been attributed, at least in part, to insufficient in utero vitamin D levels, because of low maternal serum vitamin D levels during winter [79,80]. In a large population-based case-control study [81], children born with 25(OH)D levels <10 ng/mL seemed to be at a high risk of developing MS. Likewise, the level of sun exposure in childhood and adolescence (e.g., by outdoor leisure activities), which may serve as a proxy for vitamin D supply in early life, has been inversely linked to the risk of MS in adulthood [82,83]. In a recent longitudinal Canadian study of 302 children with the acute demyelinating syndrome, low vitamin D levels were significantly associated with MS risk in the subsequent three years [84]. One report showed that children with higher serum 25(OH)D concentrations at presentation, with an acquired demyelinating syndrome, had a lower risk of early MS diagnosis [85]. Gender- and sex-related immunological differences may influence the association between vitamin D and MS. The disproportional increase in the incidence of MS in women is likely to be caused by sex-specific exposure or susceptibility to environmental factors [86]. The data supporting an interaction between female sex, possibly mediated by estrogen, and vitamin D for MS risk is accumulating. A protective effect of sun exposure was only observed in female monozygotic twins [87], and the association of sun-sensitive skin types with a disability was only found in untreated female MS patients [88]. In vitro studies of MBP-specific T cell proliferation have shown sex differences in the metabolism of vitamin D, which were confirmed by treating male MBP-specific T cells with 17β-estradiol in the assay [89]. In one animal study, vitamin D resulted in fewer clinical, histopathologic, and immunologic signs of Experimental Autoimmune Encephalomyelitis (EAE) in female mice compared with ovariectomized females and intact or castrated males [90]. A synergy between E_2_ and vitamin D occurred through the VDR-mediated enhancement of E_2_ synthesis, as well as through the E_2_-mediated enhancement of VDR expression due to the inflammation of central nervous system (CNS). In males, E_2_ did not enable vitamin D to inhibit EAE [91], possibly suggesting that vitamin D-mediated protection in EAE is female-specific, and that MS tends to have a more aggressive course in men than in women. 

Several genome-wide association studies (GWAS) and gene-candidate studies have investigated the influence of the specific genetic polymorphisms of vitamin D metabolites on the 25(OH)D levels, and their susceptibility to MS; one study found [92] an association between the short variant of the VDR protein (F allele) and a genetic predisposition to lower 25(OH)D levels, but not to a higher risk of MS; in a GWAS of 4501 European patients [93], single-nucleotide polymorphisms (SNPs) of the gene encoding components of the vitamin D binding protein were associated with 25(OH)D concentrations, or with the genes involved in vitamin D synthesis or activation; moreover, a genetically lowered 25(OH)D level was strongly associated with increased MS risk and progression in two studies [94,95].

The role of vitamin D in MS disease progression has also been assessed: it has been hypothesized that 25(OH)D levels can predict a later development of MS in acute optic neuritis (ON) [96], but the result is inconclusive. A discrete quantity of studies demonstrated that vitamin D levels affect clinical relapses and MS disease activity. In a retrospective study of 110 patients with pediatric-onset MS, the authors found that each increase of 10 ng/mL in the 25(OH)D level was associated with a 34% decrease in the relapse risk [97]. Similar findings were seen in a prospective cohort study, whose authors concluded that raising 25(OH)D by 20 ng/mL could decrease the hazard of relapse by up to 50% [98]. In a prospective longitudinal study, the relapse risk was significantly reduced in those patients with medium (20–40 ng/mL) and high (>40 ng/mL) serum vitamin D levels, compared with those with low levels (<20 ng/mL) [99]. Moreover, the same authors found that for each doubling of the serum vitamin D concentration from a baseline of 10, 20, and 30 ng/mL, MS relapse risk decreased by 27%. In another study, lower vitamin D levels predicted a conversion from Clinically Isolated Syndrome (CIS) to clinically definite MS [100,101]. In the study by Embry et al., low serum 25(OH)D levels predicted an increased likelihood of gadolinium-enhancing lesions (Gd+) in the Magnetic Resonance Imaging (MRI) scans performed in the subsequent two months period [102]. In the EPIC study [103], a five-year MS cohort study in which subjects had clinical and MRI evaluations and gave a blood sample annually. The authors concluded that individuals with CIS/relapsing-remitting multiple sclerosis (RRMS) with higher vitamin D levels have a lower risk of the subsequent development of new T2 lesions and Gd+ lesions on a brain MRI, even after accounting for potential confounding factors. Moreover, an increment of 10 ng/mL of 25(OH)D was associated with a 15% lower risk of new T2 lesions and a 32% lower risk of Gd+ lesions [103]. In a post hoc analysis, including up to two years of follow-up of participants treated with interferon beta (IFNB)-1b in the BENEFIT trial [104], Gd+ lesions development was inversely associated with 25(OH)D levels, those patients whose 25(OH)D levels were >20 ng/mL had a 39% lower risk of new Gd+ lesions. Unfortunately, across all of the analyses, associations with lower vitamin D were generally stronger for MRI than for the clinical outcomes. Moreover, the participants of the Betaferon Efficacy Yielding Outcomes of a New Dose (BEYOND) study (BEYOND study) [105], treated with IFNB-1b, with higher serum 25(OH)D levels, had lower numbers of new T2 and Gd+ lesions during the first 12 months of follow-up. Moreover, a 20 ng/mL higher serum 25(OH)D level was associated with a 31% lower rate of new lesions, and the patients with 25(OH)D ≥ 40 ng/mL showed 47% lower rate of new T2 lesions and new Gd+ lesions, when compared with the patients who had serum levels of 20–32 ng/mL. Vitamin D and disease-modifying therapies (DMTs) may positively influence each other and produce an additive, or even synergistic, effect on MS disease activity. In an observational cohort study, which included 178 patients with MS [106], the patients who were treated by IFN had significantly higher 25(OH)D levels than those who were not. Interestingly, the IFN treatment was protective only against relapses among the patients with higher vitamin D levels. The authors hypothesized that treatment with IFNB might increase the serum vitamin D levels through an enhanced responsiveness to sun exposure [106]. The same authors did not find similar associations for glatiramer acetate (GA) therapy and vitamin D. More recently, Laursen et al. [107] found that higher vitamin D levels in CIS may slow neurodegeneration evaluated by brain volume measures. In fact, they found that each 10 ng/mL increase in 25(OH)D was significantly associated with a 7.8 mL higher gray matter volume [108]. Variations in the relapse rate and the number of MRI brain lesions have shown a seasonal pattern that can be related to a variation in Ultra Violet Radiation (UVR) exposure and vitamin D status [109,110], with some exceptions [111,112]. Most cross-sectional studies have concluded a negative correlation between the 25(OH) D level and disability [103,113,114], and, interestingly, even a direct correlation between the 25(OH) D level and poorer memory performance [115]. However, the causality is considered uncertain, at the moment.

Given the previous reviewed findings, the assessment of vitamin D supplementation for a possible disease-modifying course of MS is obviously of crucial interest. Unfortunately, the current evidence does not offer a definite consensus for supplementation. Kimball et al. [115] performed a six-month safety study with escalating doses of vitamin D, and they found a significant reduction in the mean number of Gd+ lesions at the end of the study. In an open-label randomized trial, patients randomized to a vitamin D supplementation had an annualized relapse rate (ARR) significantly lower in the treatment, with a prolonged relapse-free time and with a persistent reduction in the T cell proliferation [44]. In a one year double-blind, randomized placebo-controlled trial with vitamin D3 as an add-on treatment to IFNB-1b, the MRI T2 lesion burden and the new/enlarging T2 lesions, tended to increase more in the placebo group than in the vitamin D group, however, without statistical significance [116]. A preliminary Iranian study assessed the safety and efficacy of high-dose vitamin D supplementation during pregnancy in women with MS [117]. The women in the vitamin D group had significantly fewer relapses during pregnancy, a tendency for fewer relapses up six months after delivery, and a more stable Expanded Disability Status Scale (EDSS) than those without supplementation [107]. In a longitudinal study [118], in which 170 natalizumab-treated patients were followed for one year between two winter seasons, patients with insufficient serum 25(OH)D levels at baseline (<20 ng/mL) were advised to take vitamin D supplements, and a significant inverse relationship with the ARR was found, as, for each nmol/L increase in 25(OH)D, a 0.014 decrease in ARR was observed. The double-blind, multicentre, 48-week study, named SOLAR, of supplementation of high-dose oral vitamin D has been the most extensive study to date [119]. An insignificant trend toward lower ARR in the treatment group was found in vitamin D groupersus placebo, and no statistically significant differences in the disease activity were found between the two groups. Vitamin D3 was associated with a statistically significant reduction in the combined unique lesions (secondary endpoint). The effect of the addition of vitamin D3 to IFNB-1a over 96 weeks was investigated with appropriate cognitive tests [120]. It did not show a significant trend toward a lower ARR (primary endpoint) among those patients receiving vitamin D treatment, which became statistically significant when the analysis was restricted to those who completed the study. Among the completers, there were also significantly fewer new T2 lesions in the vitamin D group. It is unclear whether the findings of these trials are related to insufficient power or other issues leading to an inability to detect a treatment effect for all outcomes [120].

Larger randomized controlled trails (RCTs) are currently underway to reveal the role of vitamin D supplementation as an add-on to IFNB therapy in the treatment of RRMS, and even CIS, patients, namely, the VITADEM study in Spain, EVIDIMS study in Germany, PrevANZ study in Australia, D-Lay-MS study in France, and the VIDAMS trial in the United States. One study investigated the cognitive effects of vitamin D supplementation on patients with MS who were treated with IFNB [121], and after the follow-up period they scored better on the Brief Visuospatial Memory test (BVMT) for delayed recall.

In conclusion, numerous studies suggest that vitamin D supplementation may benefit MS patients, although larger RCTs are needed to establish this supplementation as a standard of care for MS. Moreover, there is no consensus on the definition of ‘sufficient’ vitamin D levels in MS, and many physicians question whether people with MS should empirically be supplemented while waiting for more conclusive results of vitamin D clinical trials. In the view of the Institute of Medicine (IOM), 25(OH) D levels greater than 20 ng/mL (50 nmol/L) are sufficient. The Endocrine Society, considering skeletal and non-skeletal health, argues for levels of almost 30 ng/mL (75 nmol/L). In the MS field, numerous studies suggest that serum 25(OH)D levels of approximately 40 ng/mL (100 nmol/L) are the lower limit for controlling MRI and clinical activity. Some experts favor maintaining 25(OH)D levels between 30 and 50 ng/mL in MS patients, as immune-modulatory effects have been observed in these ranges in experimental studies. Longitudinal RCTs are needed to establish the recommended levels of vitamin D supplementation necessary to reduce the risk of MS onset and MS pathological activity. According to other authors [122], for individuals with CIS or MS and vitamin D deficiency (<20 ng/mL), an ‘attack’ supplementation with 50,000 IU/week of vitamin D2 (ergocalciferol) for eight weeks, and a subsequent evaluation of the serum level, is recommended. The continuation of this therapy until the serum 25(OH)D is higher than 30 ng/dL may be necessary. A maintenance supplementation with vitamin D3 (cholecalciferol) at 1000 to 2000 IU/day may be started when the deficiency has been corrected, and at once in case of vitamin D insufficiency (20–29 ng/mL). As current evidence also suggests both an obstetric and pediatric benefit from vitamin D against the risk of developing MS, vitamin D supplementation in children and pregnant women at risk of developing or being affected by MS should be considered [123]. According to other authors, as a goal, the general aim is 25(OH)D levels between 40 and 60 ng/mL, and most patients should take a dose that arises from 2000 to 5000 IU/day vitamin D3 [123]. Many studies have been conducted to show the potential relationships between genome-wide association studies (GWAS) and gene-candidate studies, in order to find out the specific genetic polymorphisms of the vitamin D metabolites on the 25(OH)D levels, and their susceptibility to MS and to vitamin D supplementation efficacy in MS [92,93,94,95]. Moreover, the Human Leukocyte Antigen (HLA) haplotype might influence epistasis, trans and cis effects, and parent-of-origin effects, as well as the Epstein Barr Virus (EBV) and vitamin D effects on the course of MS [124]. Le Mokry et al. (2015) identified SNPs associated with 25(OH)D level from SUNLIGHT, the largest (*n* = 33,996) genome-wide association study to date for vitamin D. Four SNPs were genome-wide significant for 25(OH)D levels, and all four SNPs lay in, or near, genes strongly implicated in separate mechanisms influencing 25(OH)D. The authors found out that the count of the 25(OH)D-decreasing alleles across these four SNPs was strongly associated with a lower 25(OH)D level. Other recent perspectives [95] confirmed the research by Ahn et al. [93]; it seems that there are strong links between the 25(OH)D levels and the genotyping of CYP2R1- and NAD-synthetase (NADSYN1-SNPs) in MS patients. The analysis revealed lower 25(OH)D concentrations in MS patients and an association of rs10766197 CYP2R1 SNP with MS risk. After stratifying the MS patients according to gender, the authors [95] found that the minor allele A of rs10766197 had a higher frequency in men in comparison with the women affected by MS. Additionally, the presence of allele A in men was associated with disease progression, assessed by EDSS and Multiple Sclerosis Severity Score (MSSS) scores [96]. The results are not univocal at all; a recent study focussed on VDR polymorphisms (Fok-I, Bsm-I, Taq-I, and Apa-I) that were genotyped by a polymerase chain reaction (PCR), followed by the restriction fragment length polymorphism (RFLP) analyses in both groups, and the serum 25(OH) D levels were determined in the MS patients by high-performance liquid chromatography (HPLC). The distribution of genotype and allele frequencies of the four VDR polymorphisms did not differ significantly between the MS patients and healthy controls, and were unrelated to the forms and the course of MS. Low serum levels of 25(OH) D were observed in MS patients, but no association was observed between the VDR and 25(OH) D levels, except for Fok-I. Moreover, the MS patients with the *FF* and *Ff* genotype had a significantly lower serum level of 25(OH) D [92]. Moreover, further studies are necessary to develop potential relationships between the genome-wide association studies (GWAS) and gene-candidate studies for determining the influence of specific genetic polymorphisms of the vitamin D metabolites on the 25(OH) D levels, and the susceptibility to MS and to vitamin D supplementation efficacy in MS.

## 3. Vitamin D Deficiencies and Ischemic Stroke

Stroke is the leading cause of significant long-term disability all over the world, and is an enormous source of global disease burden. Ischemic stroke recognizes a heterogeneous etiology, caused by not modifiable risk factors (genetic, age, and sex), and modifiable risk factors, like hypertension, diabetes mellitus (DM), dyslipidemia, sedentary lifestyle, and smoking [125].

Vitamin D deficiency is found to increase the risk of vascular disease and ischemic stroke in healthy subjects; the risk is higher for ischemic stroke than for hemorrhagic ones. Moreover, vitamin D deficiency is associated with other contributing factors for ischemic stroke (i.e., hypertension, hyperlipidemia, diabetes mellitus, and ischemic heart disease). In a stroke, vitamin D deficiency might relate with higher disease severity and adverse outcomes, including death; moreover, hypovitaminosis D is independently associated with larger ischemic infarct volume [126]; finally, vitamin D deficiency is related to slower recovery after stroke. Moreover, recently, it has been found [125,126] that lower concentrations of 25(OH) D were associated with a higher risk of incident stroke in models adjusted for age, race, age and race interaction, and sex. The magnitude and strength of the associations were unchanged after the adjustment for the season of blood draw; systolic blood pressure; BMI; diabetes; current cigarette smoking; atrial fibrillation; and use of anti-hypertensive medications, aspirin, and statins. In their work, no statistically significant differences in the association of lower 25(OH) D with a higher risk of incident stroke were observed in black subjects when compared with white subjects. Vitamin D can inhibit the development of thrombosis, which may provide a rational explanation for the relationship between vitamin D and ischemic stroke. Nevertheless, vitamin D might induce hemorrhagic stroke through other mechanisms, such as inflammation and endothelium shear stress. Even less is known regarding the relationship between vitamin D deficiency and other cerebrovascular diseases, like vessel dissection. Vianello et al. [127] reported that in acute aortic dissection (AAD), hypovitaminosis D is not associated with changes in bone-related metabolic pathways, but is inversely related to osteocalcin, which could be an exciting molecule that is able to mediate the effect of an inadequate 25(OH) D level at vascular level.

Moderate to strong associations between lower serum 25(OH)D concentrations and stroke were identified in different analytic approaches, even after controlling for traditional demographic and lifestyle covariates. Such associations were most evident among young females, younger than 50 years. The mechanisms behind the associations between vitamin D and cerebrovascular and cardiologic profiles have been widely examined in both animal and human studies. Accumulating evidence has shown adverse regulatory effects of vitamin D on the renin-angiotensin system. Renin expression was significantly increased among the vitamin D receptor knockout mice and suppressed among the wild-type mice after being injected with 1,25(OH)_2_D. The treatment of vitamin D in rats may lead to increased endothelium-dependent vascular relaxation and the inhibition of vascular smooth muscle cell growth and proliferation. Vitamin D supplementation in human subjects may contribute to improved insulin sensitivity and beta-cell function, as well as lower levels of inflammatory markers [128,129,130].

Moreover, vitamin D deficiency has been shown in several pathologies related to higher incidence of stroke. Shah Sanket et al. reported that a vitamin D deficiency was observed in a significant number of patients with chronic obstructive pulmonary disease (COPD), and in more than half of the study subjects there is an increasing frequency across the combined COPD class of cardiovascular and cerebrovascular disease [130]. Moreover, the stroke patients with enough vitamin D had more favorable outcomes, including improved muscle strength and bone density. Manouchehri et al. [131] showed that a vitamin D deficiency had an increased risk of ischemic stroke by nearly seven-fold compared to the controls. They reported a 13-fold increase in the risk of large vessel stroke, and a 4.37-folded increase for the small vessel stroke was observed. The risk of stroke increases with a concomitant deficit of vitamin D, vitamin B12, and homocysteine. Serum homocysteine, vitamin B12, and vitamin D levels are associated with baseline first-ever stroke severity, but also contribute, to some extent, to its prognosis in the early period after stroke. The early detection and management of these laboratory parameters may contribute both to primary and to secondary stroke prevention. Hyperhomocysteinemia increases the likelihood of stroke and is mostly dependent on folic acid (vitamin B9), vitamin B12, and vitamin B6 serum levels. Vitamin B12 deficiency can be detected in 10–40% of the general population and may contribute to stroke and cognitive decline. The status of homocysteine, vitamin B12, and vitamin D can go further to assess the importance of these correctable factors during the functional recovery after stroke.

The patients with a low level of serum vitamin D at the onset of the stroke showed more severe disability [126]. Qiu et al. [132] also reported a strict association between lower serum levels of 25(OH) D, and the stroke recurrence and stroke mortality at 24 months. Also, the inverse association between serum 25(OH) D levels and functional outcome in patients with acute ischemic stroke had been reported. Mortality in stroke patients is higher for those subjects <75 years old with a low serum 25(OH) D level at stroke onset [133,134,135]. Few studies have also reported vitamin D deficiency after a stroke. The mean level of 25(OH) D was lower in the subacute and chronic patients than in the healthy controls; furthermore, the level of 25(OH)D was lower in the patients with a longer duration of illness after stroke, for one month. These data suggest that it might be explained by an exhaustion of vitamin D, stored before stroke onset, and insufficient synthesis after stroke. Finally, the patients with an independent gait have higher levels of vitamin D than the immobilized patients. Restricted outdoor activity may have affected the synthesis of vitamin D after the stroke and resulted in a vitamin D deficiency.

The pathophysiology of stroke is quite complex and still not completely understood. Cerebral ischemic damage is due to the activation of several inflammatory events, including the infiltration of circulating immune cells and the activation of microglia, astrocytes, and endothelial cells [136]. In stroke individuals, the mechanism involved in vessel disease, mediated by a vitamin D deficit, might consist of a release of atherogenic pro-inflammatory cytokines, which foster atherosclerotic vascular changes and might induce plaque instability [137,138]. Vitamin D plays an additional role for the regulation of the inflammation process, through prostaglandin inhibition, reduction of mitogen-activated protein kinase (MAPK), and by reducing the expression of the nuclear factor kappa B (NF-κB) pathways [41,139]. Moreover, the down-regulation of pro-inflammatory cytokines, such as tumor necrosis factor (TNF)-α, interleukin (IL)-6, IL-12, and interferon (IFN)-γ, and the up-regulation of anti-inflammatory T regulatory (Treg) and Th2 cells and their cytokines have been reported [140,141]. Thus, the deficit of vitamin D is responsible for endothelial dysfunction [142] and is considered an independent risk factor for the occurrence of acute ischemic stroke [143,144]. As previously reported, VDD is strictly associated with higher levels of high sensitivity C-reactive protein (hsCRP), remarking the anti-inflammatory activities of vitamin D, including the inhibition of IL-6 synthesis by monocytes. CRP acts on endothelial cells inducing tissue factor expression, and promotes smooth muscle and endothelial cell proliferation [145]. Moreover, CRP increases the plasminogen activator inhibitor–1 expression [146] and induces several inflammatory genes via NF-κB activation in human endothelial cells [147]. Usually, vitamin D deficiency in stroke patients preceded stroke, and the prevalence of vitamin D deficiency is more evident in stroke patients than general medical patients. On the other hand, vitamin D supplementation in post-stroke patients may play a role in the prevention of recurrent stroke and improves the functional outcome after stroke [147,148,149,150].

Zhao-Nan Wei reported that a vitamin D deficiency is associated with a 3.2-fold increased risk of poor functional outcome events. The adjustment for established cardiovascular risk factors, including glucose level, age, and National Institute of Health Stroke Scale (NIHSS) score, did not attenuate this association. For the vitamin D deficiency, the adjusted risk of mortality increased by 290% [151]. A high dose oral vitamin D supplementation produced a short-term improvement in the endothelial function in stroke patients, with a better management of blood hypertension. Moreover, a possible cardiovascular protective role of vitamin D has been proposed, in reducing the mortality risk in patients with renal failure.

A meta-analysis of 50 RCTs of supplemental vitamin D, administered for a median of two years, involving predominantly senior women who were mainly in institutions and dependent care, showed that vitamin D decreased mortality [152]. Moreover, vitamin D3 (cholecalciferol) has been shown to decrease mortality significantly. Gupta et al. [152], in a randomized controlled open-label trial, found that patients with acute ischaemic stroke that received vitamin D and calcium supplementation, along with the usual care, compared with those receiving usual care alone, presented a higher probability of survival, achieving a better outcome at six months compared to the controls [153]. Concerning stroke, the VITamin D and Omega-3 triaL (VITAL) studied the effect of supplementation on the total cancer and major cardiovascular events (a composite of myocardial stroke, and death due to cardiovascular events), thus reporting significant effects only on a reduction of bone fractures [154,155]. Similarly, the benefit of vitamin D supplementation for stroke-related depressive symptoms is still being debated [156]. If vitamin D contributes to post-stroke recovery by pathophysiological mechanisms that should still be elucidated (some could be related to a reduction in the volume of the cerebral infarct and to neuroprotective properties), vitamin D supplementation could bring the hope of a benefit that is not known yet.

In summary, the precise relationship between vitamin D and stroke is still unclear, a revision of the data suggests that vitamin D status is associated with ischemic stroke and with the injury volume. The vitamin D levels should be measured in all stroke patients and should be considered as an independent stroke risk factor. The supplementation of vitamin D could be considered as a fundamental part of stroke therapy, but new studies should be done.

## 4. Vitamin D Deficiency and Neurodegenerative Diseases

Different important studies have implicated amyloid beta accumulation, hyperphosphorylation of tau, oxidative stress, mitochondrial dysfunction, and inflammation as the major responsible factors for a neurodegenerative process, which underlies Alzheimer’s disease (AD) [157,158]. Nevertheless, the calcium excitotoxic hypothesis and glutamate currents theories can support and amplify the Alzheimer’s cascade of events [159]. Moreover, many doubts still merge, especially for sporadic AD cases pathogenesis. It has been established that the etiology of the sporadic AD might involve multiple gene-environment relationships, and probably many epigenetic mechanisms [158,160]. The most debated aspects are those concerning the reasons for Abeta accumulation and the tauopathy consequences in the aging brain being seriously possible, and the relationship between their accumulation and various environmental risk factors, which the brain gathers throughout its life. Epigenetic modifications can act as first hits, with a consequence that remains latent for many years, until a second hit (probably determined by metabolic factors, i.e., altered nutrition, pro-inflammatory cytokines, and aging by itself) can promote the degenerative progression [161]. As a result of these premises, VDR, the major effector of vitamin D, polymorphisms have been studied in AD [162]. It has been demonstrated that there is a link between the altered gene expression of VDR and 1,25MARRS (membrane-associated rapid response steroid-binding); this association endorses the less active employment of vitamin D inside neurons, and lets them be more prone to degeneration [163]. Brewer et al. [163] and confirmed by Thibault et al. [164] recognized that cultured hippocampal cells treated with adequate concentrations of VDH (1–100 nM) were protected against excitotoxic insults, probably because of a modulation of L-type voltage-sensitive calcium channels, whose increase has been documented in hippocampus aged cells [165], and the aged long-term cultured hippocampus cells [166]. VDH down-regulates mRNA expression for different subunits of L-type voltage-sensitive calcium channels. Consequently, VDH has a fundamental role in the homeostasis of calcium-mediated activities [164], such as neuronal death and apoptosis. The VDR^−/−^ knock-out model shows a higher sensitivity to neuro-degeneration, with a rapid increase of calcium currents and neural death [166,167]. Many different VDR polymorphisms have been described as increasing the susceptibility to AD [168], mainly due to the altered expression of neurotrophins. Genome analyses, transcriptomics, and proteomics have pointed out the role of VDR polymorphism in late-onset AD susceptibility [162]. In fact, even in animal models, vitamin D seems to interfere with cognitive functions, even in other ways, probably through its different polymorphic gene expression of VDR [26]. It seems, for instance, that Bsm I and Taq I altered carriers are more prone to manifest memory and cognitive dysfunctions, as well as a vitamin D defect; on the contrary, the APA-I haplotype is associated with an increased risk of fractures, but not memory alterations [169]. Therefore, in line with Buell and Dawson-Hughes [169], calcium concentrations are not likely to vary in the different haplotypes, indicating a protective effect on the brain, beyond the calcium homeostasis. Thus, in the Alzheimer disease culture models, VDH stimulates the amyloid plaques [170,171], supporting the phagocytosis induced by macrophages of a soluble amyloid beta protein [171,172], and reduces the inflammation response induced by amyloid deposition [21]. Lipopolysaccharide-induced levels of mRNA encoding for macrophage colony stimulating factors and tumor necrosis factors in cultured astrocytes are partially reduced after vitamin D treatment [21]. Additionally, vitamin D has neuroprotective properties against glutamate toxicity [173]. It inhibits the synthesis of inducible nitric oxide synthase and regulates the gamma-glutamyl transpeptidase, fundamental in the metabolism of glutathione [174]. Furthermore, vitamin D enhances the protein phosphatase 2A activity, modulating the redox state, and thus reducing the age-related tau hyperphosphorylation, limiting the cascade of neuronal dying back and the promotions of collateral inflammatory potentiation [175]. To summarize, it seems that vitamin D acts in the brain, through the regulation of NGF and neurotransmitters, regulating calcium homeostasis, promoting anti-inflammatory responses, interfering with amyloid beta metabolism, and implementing a brain oxidative response. On the contrary, clinical practice does not give univocal results. A vitamin D deficit has been widely detected in the frail old population [176,177], and calcium homeostasis is heavily lowered in neurodegenerative pathologies, such as Alzheimer disease [177,178]. In a mild cognitive impairment population, not including Alzheimer disease, a low level of vitamin D was reported [179]. Nevertheless, the vitamin D implementation in clinical practice does not give univocal results. A seven-year follow-up study confirmed that a larger intake of vitamin D is associated with a lower risk of developing AD in normal aging women [180,181]. Some recent works have evidenced that the combined effect of vitamin D and docosahexaenoic acid can enhance the neural protection towards the different effects of beta-amyloid deposition [182,183], and a six-month trial determined that the patient obtains better results, with even limited effects, when the memantine was prescribed in association with vitamin D, rather than alone, in the AD patients [182]. On the other hand, two studies showed the opposite results [183,184]. Even though many different studies are needed to demonstrate a definite role in the real clinical practice of the supplementation of vitamin D, many questions remain without a proper answer, such as:

1. When should the implementation begin?
2. Is it a possible preventive therapy for reducing the cascade of events of the AD?
3. Should it be considered as one of the multifactor agents (along with folate, vitamin B12, and antioxidant substances) that can procrastinate the AD second hit process?

### 4.1. Vascular Dementia

Emergent evidence from experimental and clinical studies suggests that vitamin D may be a causal effect for neurodegeneration, concerning vascular dementia, supporting the underlying/concomitant atherosclerotic pathology [185]. Some studies link the vitamin defect to an increased risk of hypertension, diabetes, congestive heart failure, myocardial infarction, and stroke [186]. Moreover, some very recent studies link it to small vessel disease and vascular dementia as well [187,188]. The vitamin D receptors are hugely expressed by the endothelial cells, and their activation induces the promotion towards the maturation of immature cells, via Vasoactive Endothelium derived Growth Factor (VEGF) [189]. The vitamin D receptor gene is up-regulated during inflammation in endothelial cells [190,191,192], and the vitamin D analogs protect against advanced glycation products derived insults [193]. Enriched vitamin D diet models promote an anti-lymphoproliferative effect on the endothelium and a diminished response to inflammatory cytokines [194,195]. Moreover, as previously described, vitamin D regulates the expression of 74 genes and 36 proteins, connected with the correct development of the cytoskeleton and exerting a regulation on post-transcriptional controls for L-type voltage-sensitive calcium channels [196,197]. All of the effects of vitamin D on the vascular system link it to vascular dementia in many different ways, such as: epidemiological [198], based on vascular pressure control [191], based on biological properties of vitamin D, above described [199], or simply considering vitamin D deficiency as a risk-modifiable factor [170,200,201]. It seems quite interesting that the three works that link the vitamin D defect to small vessel disease-related dementia [188,189] stand on two axiomatic biological properties, the anti-oxidative capacity of vitamin D (therefore a loss of protection against Reactive Oxygene Species (ROS), whenever it lacks), and the control of the smooth vessel, which is fundamental for auto-regulation in brain circulation. Many studies evidenced an excess of superoxide by Nicotinamide Adenine Dinucleotide Phosphate Hydrogen (NADPH) oxidase in small vessel disease [202]. This causes an increase of ROS, indirectly evidenced by a hyper-expression of the Nitrogen Oxide (NOX) 2 and NOX 4 oxidase isoforms [202,203]; ROS induced damage is one of the triggers for apoptosis. In vitro, vitamin D down-regulates the activity of NF-κB activity [204] and stimulates the anti-inflammatory cytokines [205]. Additionally, vitamin D-binding proteins are more evident in proximity to endothelium injury [206], inhibiting the expression of Matrix Metalloproteinase (MMP-2, MMP-9), and of the endothelium growth factor [207]; they probably diminish the activity of the platelet-derived growth factor, conversely up-regulating thrombomodulin [208,209]. The second study, entirely dedicated to small vessel disease and vitamin D [198], lights some shadows in a fascinating problem; it presents a public association between bone small vessel disease and osteoporosis. Authors hypothesize a common method of alteration, in the peripheral autonomous nervous system, which deteriorates the small-bone vessels as well as brain vessels [210]. The first and the third study on the topic [188,189], starting from two different perspectives have some common points. Chung et al. [188] demonstrated that vitamin D is inversely associated with lacunes, white matter hyperintensities, and deep micro-bleeding in the deep white matter, not elsewhere, suggesting a high impact on small vessel disease. Moretti et al. [198] hypothesized that vitamin D might act on the altered control of cerebral blood flow (CBF) [211,212] and in the distorted neurovascular coupling system [213,214], both heavily impaired in small vessel dementia. Intracerebral calcium, intimately regulated by vitamin D, interferes with the vessel activation. Vessel relaxation is heavily influenced by ATP-sensitive potassium channels (delayed rectifier and inward rectifier potassium channel), but also by calcium-activated potassium channels [215,216]. Mediated by cAMP, calcium-activated-potassium channels seem to be involved in the negative “feedback system to regulate vascular tone” [215]. It seems that in atherosclerosis models, there is a major impairment of calcium-activated potassium channels in mainstream vessels [215]. On the other hand, the neurovascular coupling system (smooth vascular cells, neurons, and astrocytes) seems to be intimately regulated by vitamin D, which is determinant for the glutamate release. Glutamate passes from the synapse, activating Nicotinamide Adenine Dinucleotide Phosphate Hydrogen- Receptors (NMDAR) in the neurons and metabotropic glutamate receptors in the astrocytes [214]. Moreover, by interfering with the calcium influx, vitamin D indirectly mediates the neural nitric oxide synthase. NO activates phospholipase A2 in the astrocytes, enabling the prostaglandin cascade, which widens the arteries. The presence of vitamin D increases, moreover, acetylcholine, and VIP, which potentiates the mechanism. Low levels of vitamin D might, therefore, interfere with smooth cells control, with NO and neuropeptides synthesis, and with defective neurovascular coupling, leading to some aspects of small vessel disease. There are very few the studies concerning vitamin D supplementation in vascular dementia.

### 4.2. Parkinson Disease (PD)

A significant concentration of vitamin D receptors were found in the hippocampus, in the prefrontal cortex–brain, and the substantia nigra [217]. There is evidence of low levels of vitamin D and increased bone turnover markers, such as bone alkaline phosphatase, compared to the controls [218]. All of the non-genomic roles of vitamin D are evident in the PD models [219,220]; there is an active regulation of calcium inside the neurons and astrocytes; there is a substantial regulation of L-type voltage-sensitive calcium channels; it has been described an induction and promotion of NGF and other growth factors, with a down-regulation of MMP and other inflammatory activity, such as prostaglandins and COX-2 activity; and there is also a reduction of oxidative stress, by interfering with NO production by lipopolysaccharide-stimulated macrophages [221]. There are many data that even relate the vitamin-related genomic factors to PD progression. Increased levels of MHC class II expressions were detected in the monocytes of the cerebrospinal fluid of the PD patients [222], together with Human Leukocyte Antigen Complex- antigen D related (HLA-DR) positive reactive microglia, found in the *substantia nigra* and in nigro-striatal tract of PD patients [223]. Recent evidence demonstrated that vitamin D suppresses the MHC class II antigens [224] and IFN-gamma induced HLA-DR antigen expression in human cells [225]. Additionally, Cytochrome P450 (CYP), and in particular, CYP2D6, which has a polymorphic expression, and which is expressed in neurons and the gut, has a different expression in PD patients; here, it has been found preponderantly as the CYP2D6*4 allele [226]. CYP2D6 acts as a 25-hydroxylase, which can convert vitamin D3 into 25OHD, being the key enzyme to determine a deficiency of vitamin D. In the mouse MPTP treated model (inducing PD phenotype), Singh et al. [225] reported the stable expression of the animal ortholog of CYP2D6, CYP2D22. Very interestingly, the CYP2D loci are located on chromosome 22, where many genes related to PD are segregated [227,228]. It seems surprising that the deletion of chromosome 22q11 was reported to be associated with PD [228], but also with the reduced serum calcium and a reduced level of vitamin D [228].

Moreover, there are three more links between vitamin D and PD. The first one is the so-called Sp1 transcription factor. The protein encoded by this gene is a zinc finger transcription factor that binds to the guanine-cytosine (GC)-rich motifs of many promoters. The encoded protein is involved in many cellular processes, including cell differentiation, cell growth, apoptosis, immune responses, response to DNA damage, and chromatin remodeling. Post-translational modifications, such as phosphorylation, acetylation, glycosylation, and proteolytic processing, significantly affect the activity of this protein, which can be an activator or a repressor. The Sp1 transcriptions factor is a DNA binding protein that mediates the accurate response to oxidative stress in neurons [229,230], controls the expression of the dopamine transporter gene, and of the dopamine receptor gene [231,232]. The link between vitamin D and Sp1 is represented by the fact that many hormones have different binding sites for the transcription factors for Sp1. In fact, two vitamin D-responsive elements (which act by inducing the expression of CYP24 genes, previously described, and 25-OHD-24 hydroxylase) are helped by the synergic activation of Sp1 [229,233]. As Sp1 controls the expression of the dopamine transporter gene within the dopaminergic neurons, VDR conditions and promotes Sp1 trigger gene expression.

The second genomic link factor between vitamin D and PD is represented by Heme oxygenase, a stress protein with anti-oxidant properties. In the healthy brain, its expression is limited to neuroglia [234], whereas it is overexpressed in PD brains, but not in AD patients [235]. In PD brains, Heme oxygenase 1 is overexpressed in astrocytes within the *substantia nigra* and the deteriorated dopaminergic neurons [236]. Entirely unpredicted, calcitriol seems able to delay the effect of Heme oxygenase, reducing the glial fibrillary immunoreactivity [237].

Finally, vitamin D seems to interfere with the activity of Poly (ADP-Ribose) Polymerase-1, also called PARP1. It is a stress protein, which can, however, promote neuronal death. In fact, there is an overexpression of PARP1 in the *substantia nigra* of PD patients [238]; increased levels of vitamin D down regulate the PARP-1 expression, probably mediated by a diminishment of microglial activation [239]. All of these aspects considered in the clinical trials on vitamin D supplementation in Parkinson’s disease have been carried out. Although some data demonstrated that low levels of vitamin D might influence the speed of the disease, the supplementation of vitamin D did not modify the disease outcomes. That conclusion probably lies in a clinical time of supplementation, far from the initial ones of labs models.

## 5. Conclusions

Accumulating evidence shows that vitamin D is important for accurate brain functioning. Moreover, it is important to note that many important neurological diseases are related to low levels of vitamin D. These expressions are supported by in vivo, in vitro, and in human trials. Unfortunately, at the moment, the results for its supplementation in neurological disorders, in particular concerning data on vitamin supplementation and their progression or eventual real clinical benefits are inconclusive. As such, there is a strong need for randomized clinical trials on neurodegenerative patients and vitamin D supplementation, to optimize time, efficacy, and appropriate monitoring [240,241].

It should be said that vitamin D supplementation might be safe and inexpensive. However, physicians may be uncomfortable recommending larger doses of vitamin D, because of its potential toxicity. The rarity of reports of vitamin D toxicity can be explained, in part, by the kidney’s ability to limit the production of active calcitriol. Increased calcitriol levels inhibit parathormone (PTH), causing the calcitriol production in the kidney to decrease. The renal 24-hydroxylase activity further limits the availability of calcitriol by creating inert metabolites of both calcitriol (1,24,25-trihydroxyvitamin D) and calcidiol (24,25-dihydroxyvitamin D). The 24-hydroxylase gene is under the transcriptional control of calcitriol, thereby providing a tight negative feedback.

Concerning vitamin D replacement, several studies have shown that both vitamin D2 and vitamin D3 are effective in maintaining the serum 25(OH)D levels. Both D2 (ergocalciferol) and D3 (cholecalciferol) are available as dietary supplements, and both appear to be effective. A single dose of 50,000 IU of D2 or D3 produces a similar increase in the total 25(OH)D concentration, but the apparent longer half-life of D3 suggests that less frequent dosing may be needed. To replenish the serum 25(OH)D levels in persons with a vitamin D deficiency, one cost-effective regimen is oral ergocalciferol at 50,000 IU per week for eight weeks. The optimal time for rechecking the serum levels after being replenished has not been clearly defined, but the goal is to achieve a minimum level of 30 ng per mL.

Special mention is needed for patients who require tube feeding or parenteral nutrition. In those cases, ergocalciferol capsules containing D2 in oil, which can clog the feeding tube, should not be used. Cholecalciferol capsules and tablets contain D3 in powder form and can be used.

New approaches are emerging in vitamin D deficiency. The combined treatment using bisphosphonates and vitamin D has been suspected to be more effective compared with bisphosphonate or vitamin D alone in the prevention of atherosclerosis. In a recent study, the combination of bisphosphonates and vitamin D has been seen to be safe and effective in systemic lupus erythematosus. Bisphosphonates are expected to inhibit the arterial plaque development and calcification through several mechanisms (mostly inducing decreased levels of inflammatory cytokines and matrix metalloproteinases). Bisphosphonates also decrease a variety of mature vascular cells, which migrate into the vessel walls and injure vascular endothelial cells. Moreover, treatment with statins in combination with bisphosphonates has been shown to be more effective in terms of reducing atherosclerotic plaque, compared with either monotherapy in patients with hypercholesterolemia [242,243].

Reading what the literature has reported, it seems that the supplementation of vitamin D might reduce the risks and improve the pathological features in different neurological conditions, but, because of different and unknown reasons, it seems that its supplementation might not be sufficient to change the outcomes and the disease phenotype. All of this considered, there is a strong need for further research in this field, even to account for the best quality of vitamin implementation.
